# A Nonlinear Dynamics Approach for Incorporating Wind-Speed Patterns into Wind-Power Project Evaluation

**DOI:** 10.1371/journal.pone.0115123

**Published:** 2015-01-24

**Authors:** Ray Huffaker, Marco Bittelli

**Affiliations:** 1 Agricultural and Biological Engineering Department, University of Florida, Gainesville, Florida, United States of America; 2 Department of Agricultural Sciences, University of Bologna, Bologna, Italy; Centro de Investigacion Cientifica y Educacion Superior de Ensenada, MEXICO

## Abstract

Wind-energy production may be expanded beyond regions with high-average wind speeds (such as the Midwest U.S.A.) to sites with lower-average speeds (such as the Southeast U.S.A.) by locating favorable regional matches between natural wind-speed and energy-demand patterns. A critical component of wind-power evaluation is to incorporate wind-speed dynamics reflecting documented diurnal and seasonal behavioral patterns. Conventional probabilistic approaches remove patterns from wind-speed data. These patterns must be restored synthetically before they can be matched with energy-demand patterns. How to accurately restore wind-speed patterns is a vexing problem spurring an expanding line of papers. We propose a paradigm shift in wind power evaluation that employs signal-detection and nonlinear-dynamics techniques to empirically diagnose whether synthetic pattern restoration can be avoided altogether. If the complex behavior of observed wind-speed records is due to nonlinear, low-dimensional, and deterministic system dynamics, then nonlinear dynamics techniques can reconstruct wind-speed dynamics from observed wind-speed data without recourse to conventional probabilistic approaches. In the first study of its kind, we test a nonlinear dynamics approach in an application to Sugarland Wind—the first utility-scale wind project proposed in Florida, USA. We find empirical evidence of a low-dimensional and nonlinear wind-speed attractor characterized by strong temporal patterns that match up well with regular daily and seasonal electricity demand patterns.

## Introduction

Penetration of wind power into power grids is challenged by intermittent and fluctuating wind speeds restricting the dispatchability of wind power to meet daily and seasonal patterns of energy demand. Wind-speed fluctuations over land follow prominent diurnal patterns resulting from thermal heat exchanges with oceans [1,2]. Wind-power penetration can increase with less reliance on costly grid-scale electricity storage systems if diurnal patterns produce top wind speeds during daily peak energy demand hours. Favorable matching of wind-power supply and energy-demand patterns also might profitably extend wind projects to sites with relatively low average wind speeds [3]. Consequently, there is critical need in project evaluation to model wind-speed dynamics accurately reflecting real-world patterns [1,3].

The conventional method for incorporating wind-speed patterns into project evaluation is to remove and then restore them synthetically. Patterns are removed as random-appearing observed wind speeds are modeled probabilistically. Wind speeds are defined as a set of random variables governed by an explicit probability distribution—typically the Weibull for its simplicity and success in fitting wind speed data [2,4–10]—or a set of random processes governed by probability transition matrices. Wind-speed patterns are synthetically restored to probabilistic frameworks because removed patterns are informative to project evaluation, and failure to account for temporal correlations results in statistical bias [2,11].

The search for restoration methods has been vexing due to the difficulty of synthesizing wind-speed patterns adequately approximating real-world complexity. One- and two-step Markov chain models have difficulty capturing low-frequency diurnal wind speed dynamics. Embedded Markov chain models with two probability transition matrices separating high-frequency from low-frequency behavior must still choose between adequately representing seasonal or diurnal variability in the low-frequency matrix (see [3] for an extensive review). The most recent contribution to the probabilistic energy planning literature further refined synthesis of natural diurnal and seasonal patterns by: (1) modeling transitions among “non-peak” day-types (days without significant wind variability) and “peak” day-types (each having maximum wind energy available in different 6-hour blocks during the day); and (2) fitting a sine-curve to monthly mean wind speeds [3]. Progressively complicated pattern restoration methods substantially increase the conceptual and computational complexity of proposed planning frameworks.

We propose a paradigm shift in wind-power evaluation to empirically diagnose whether synthetic pattern restoration can be avoided altogether. If complex behavior of observed wind-speed records is due to nonlinear, low-dimensional, and deterministic system dynamics as opposed to uncontrolled and inherently random processes, then nonlinear dynamics methods can reconstruct wind-speed dynamics from observed wind-speed data without prior knowledge of system dynamics [12], and without recourse to probabilistic approaches removing temporal structure. Nonlinear time series analysis is a recognized empirical method for analyzing climate variability [13]. In particular, low-dimensional nonlinear wind-speed dynamics were recently reconstructed from a ten-year record of daily mean wind speeds in India [14]. Nonlinear dynamic analysis is an untested approach in wind power evaluation.

We test a nonlinear dynamics approach in an application to Sugarland Wind—the first utility-scale wind project proposed in Florida, USA. The proposal is for a 200 megawatt wind farm with 114 turbines spread across 12,887 acres of sugar cane fields in the Everglades Agricultural Area [15]. It is located in the Southeastern USA—a region where wind power is undeveloped due to insufficient wind [16,17]. An industry publication referred to Sugarland Wind as “another can’t be done” project now feasible due to breakthroughs in turbine technology capable of generating commercially viable power with lower wind speeds [18]. We investigate the extent to which wind power patterns correspond with energy demand patterns, and thus potentially compensate for lower mean wind speeds in increasing commercial viability of Sugarland Wind. We find empirical evidence of a low-dimensional and nonlinear wind-speed attractor characterized by strong temporal patterns that match up well with regular daily and seasonal electricity demand patterns.

**Fig 1:**
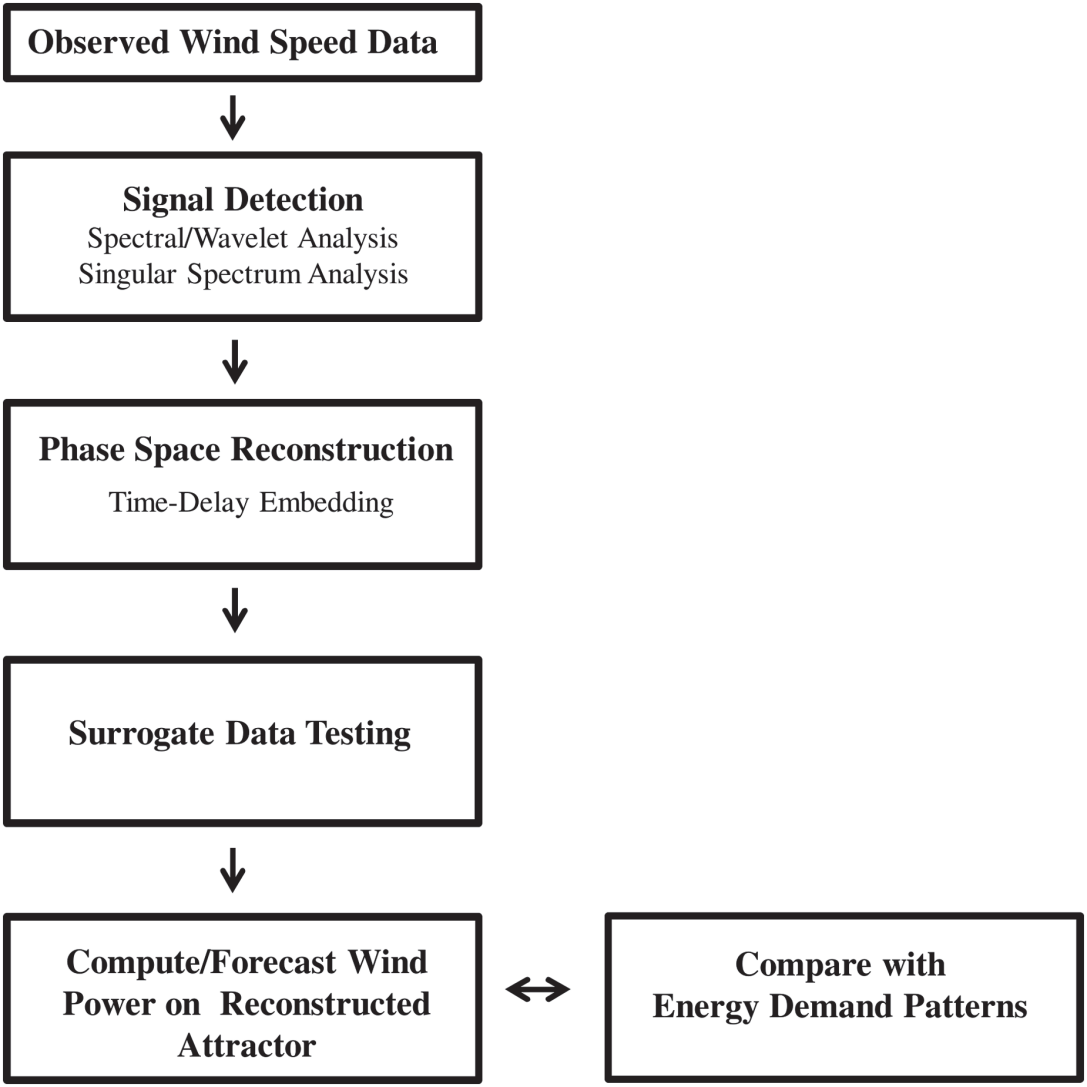
Proposed procedure for integrating nonlinear dynamic methods into wind project evaluation.

A four-stage procedure for integrating nonlinear dynamic methods into wind project evaluation is summarized in Fig. 1. In Stage 1, signal detection methods separate structural content from noise in the data. Spectral analysis identifies periodic patterns in the data, and wavelet analysis tests for spectral stationarity required to apply Singular Spectrum Analysis (SSA) [1]. SSA decomposes the time series into the sum of structural (trend and oscillations) and unstructured-residual components. In Stage 2, the SSA structural component is used to reconstruct system dynamics generating observed wind speed data (‘phase space reconstruction’) [13,19]. In particular, we investigate whether real-world wind speed dynamics evolve along a low-dimensional attractor. An attractor exists when system dynamics pull initial conditions toward a spatially-organized structure upon which system variables either remain constant (a ‘point’ attractor), orbit periodically (a ‘limit cycle’ attractor), or orbit irregularly (a ‘strange’ attractor) as time approaches infinity. In Stage 3, surrogate data are generated to test the null hypothesis that the attractor’s apparent structure is the figment of a mimicking stochastic process [20,21]. In Stage 4, wind power produced by wind speeds along the reconstructed attractor is calculated to determine how well supply patterns coincide with available information on peak daily energy demand patterns. State space forecasting methods are applied to make short-term predictions of wind power—a primary duty of wind project evaluation.

## Data

### Daily Electricity Demand Loads

**Fig 2:**
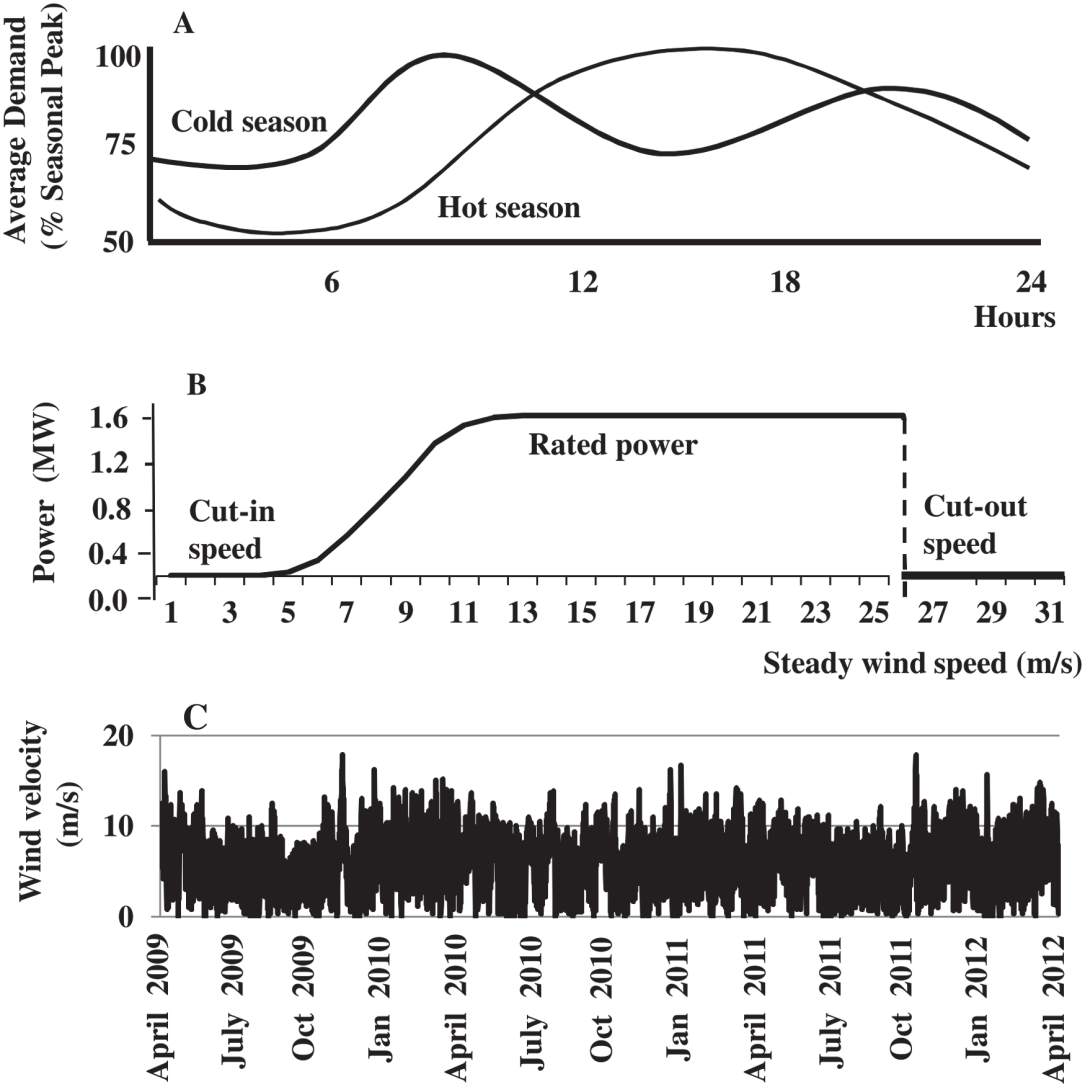
Data. (A) The typical daily electric demand load for hot and cold seasons. (B) Power curve for General Electric 1.6–100 turbine. (C) Preprocessed six-hour wind-speed record from April 1, 2009 through March 31, 2012 (4380 observations).

Daily electricity demand loads fluctuate hourly and seasonally in the project area [22] (Fig. 2A). Demand is generally low during nighttime sleeping hours, rises in morning hours as people start their day, and remains high throughout business and evening hours as people engage in household chores and entertainment. During the hot-season, electricity demand increases through the day due to increasing air conditioning loads brought on by high temperatures and humidity, and peaks in late afternoon. During the cold-season, demand peaks in mid-morning and early evening due to heating loads. Winsberg and Simmons [23] estimate average hot and cold seasons for the project area to be roughly April-October and November-March, respectively.

### Electrical Power Generation

Turbines convert wind’s kinetic energy into mechanical energy that turns a rotor to generate electricity. Power produced by turbines on a wind farm tends to be mutually uncorrelated due to turbine spacing and surface roughness [1]. We further assume that turbines in the Sugarland Wind project operate under identical wind conditions, and thus limit our attention to a single turbine without loss of generality. The Sugarland Wind developer is adopting a General Electric 1.6–100 turbine for similar scale projects currently under construction [24]. The GE 1.6–100 has a hub elevation and rotor diameter of 100 meters (*m*) [25]. The turbine begins to produce electrical power at a ‘cut-in’ wind speed of 3.5 meters/second (*m/s*), produces a maximum ‘rated-power’ output of 1.6 megawatts (*MW*) at a ‘rated-power’ wind speed of 12 *m/s*, and continues to produce rated-power output up to a ‘cut-out’ speed of 26 *m/s* when the turbine shuts down to avoid wind-related damage [10] (Fig. 2B).

### Wind Speed

The record of hourly surface wind speeds from April 1, 2009 through March 31, 2012 for the study area (West Palm Beach/IN) was downloaded from the online National Climatic Data Center (NCDC). The NCDC data were preprocessed to obtain consistent hourly reporting intervals by deleting observations taken at times other than the 53^rd^ minute of each hour (26,280 observations remain). These surface wind speeds (*v*_*s*_) were preprocessed further to approximate wind speed at hub elevation (*v*_*H*_) using the log wind profile for neutral atmospheric conditions [26]:

$$v_{H}=v_{S}\frac{\text{log}\left(el_{H}/\text{z0}\right)}{\text{log}\left(el_{S}/\text{z}0\right)}$$ (1)

where *el*_*H*_ is hub elevation (*m*), *el*_*S*_ is elevation of the NCDC measurement (10 *m*), z0 is surface roughness (~0.15 *m* for young sugar cane up to 0.3 *m* [27]), and Eqn. [1] is the ratio of the log wind profile for two wind speeds and elevations. Finally, the data were aggregated into six-hour blocks (0:00–6:00, 6:00–12:00, 12:00–18:00, 18:00–24:00) compatible with daily energy demand patterns in Fig. 2a (4380 observations). The preprocessed wind-speed record (*v*_*H*_) is highly fluctuating, variable, and without readily apparent structural patterns (Fig. 2C).

## Methods and Results

### Signal Detection

**Fig 3:**
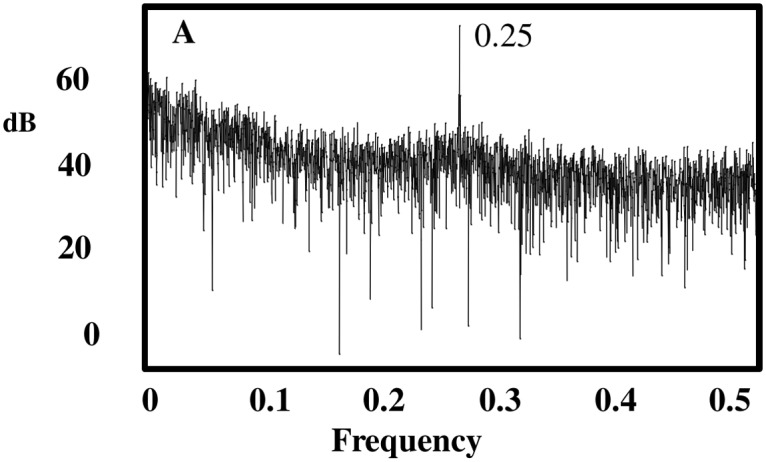
Signal Detection. Fourier power spectrum of preprocessed wind velocity series showing dominant frequency associated with a diurnal oscillation (0.25 Hz).

The Fourier power spectrum for the preprocessed wind-speed record (*v*_*H*_) exhibits a dominant peak frequency of 0.25 Hz corresponding to a 24-hour oscillation period (i.e., four 6-hour blocks) (Fig. 3). We applied Singular Spectrum Analysis (SSA) to deconstruct *v*_*H*_ into a sum of time series’ representing trend, the diurnal oscillation identified in the Fourier power spectrum analysis, and unstructured-noise [19,28,29].

SSA proceeds in three steps: decomposition, grouping, and reconstruction. In decomposition, *v*_*H*_ is embedded into the *L*×*K* ‘trajectory matrix’, *X*, whose columns are *K* = *N*—*L* + 1 single-period lagged vectors of *v*_*H*_, *N* is record length, and *L* is ‘window length’ restricted by 2 ≤*L* ≤ *N* / 2 and conventionally selected proportional to the dominant spectral peak in the Fourier spectrum [29]. Accordingly, we set *L* = 400, which allows for 100 repetitions of the diurnal oscillation period (i.e., four six-hour blocks). Next, the singular value decomposition is taken of the trajectory matrix: $X=\sum_{i=1}^{r}X_{i}$, where $X_{i}=\sqrt{\lambda_{i}}PC_{i}EV_{i}{}^{T}$ is the *i* ^th^ ‘empirical orthogonal function’ (*EOF*_*i*_), and *r = rank X*. The eigenvalue (λ_*i*_), *K*×1 eigenvector (*EV*_*i*_), and *L*×1 principal component (*PC*_*i*_) are drawn from the eigensystem of the *K*×*K* covariance matrix, *XX* ^*T*^.

In the grouping step, *EOFs* are clustered into groups forming the basis for trend, oscillatory, and unstructured residual components. The first of two grouping diagnostics is to inspect the ‘eigenspectrum’ plotting square roots (‘singular values’) of *λ*_*i*_(*i* = 1, …, r) in rank order from the largest to the smallest in magnitude. Singular values on the initial steeper portion of the eigenspectrum are associated with *EOFs* potentially forming the basis of the deterministic signal, while singular values on the remaining flat portion (‘noise floor’) are associated with ‘white noise’. The largest singular value is typically associated with slow-moving trend. Pairs of singular values that are equal (creating steps in the eigenspectrum) are associated with *EOF* pairs potentially forming the basis of harmonic oscillations. The second diagnostic is to certify that: (1) plots of the eigenvectors associated with these *EOF* pairs oscillate with identical frequency in phase quadrature; and (2) pairwise scatterplots of these eigenvectors are visually similar to pairwise scatterplots of pure sine and cosine functions oscillating at the same frequency as the paired eigenvectors. The scatterplots of eigenvectors associated with harmonic oscillations result in polygons whose number of sides indicate oscillation period. For example, a square shape indicates a four period oscillation [29].

In the reconstruction step, ‘diagonal averaging’ of grouped *EOF* matrices converts them to vector time series’ of corresponding trend, oscillatory, and unstructured residual components. A final diagnostic is to certify ‘separability’ of reconstructed time series’ as indicated by near-zero *w-correlation* coefficients [28].

**Fig 4:**
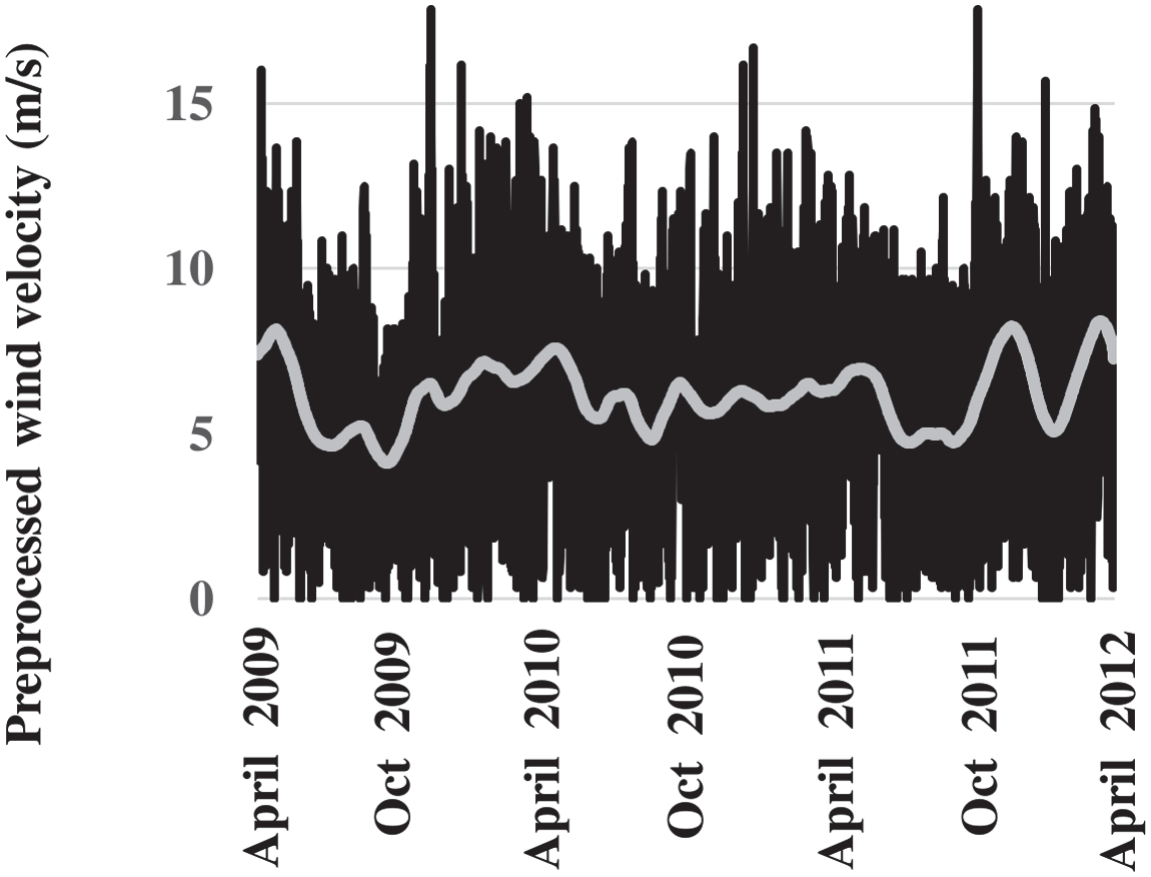
1^st^ Stage SSA. Plots of preprocessed wind velocity record (black line) and extracted trend series (grey line) reconstructed from *EOFs* 1, 4, and 5.

SSA can be applied sequentially to a time series if, for example, a complex trend component in the 1^st^ stage obfuscates identification of harmonic components in an initial application [28]. This was the case for the preprocessed wind-speed record (*v*_*H*_). First stage application extracted a trend component composed of the 1^st^, 4^th^, and 5^th^ *EOFs*. The trend time series reconstructed from this *EOF* grouping is relatively complex due to slow-moving oscillations (Fig. 4).

**Fig 5:**
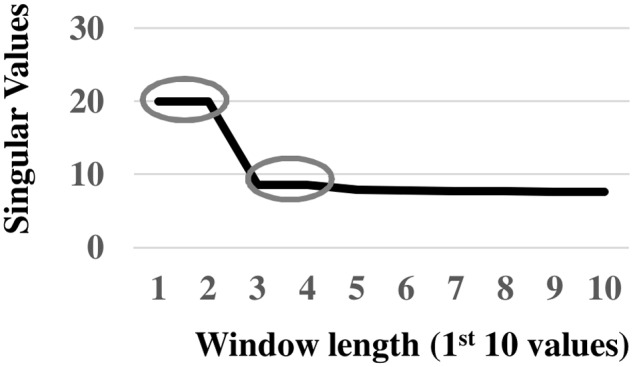
2^nd^ Stage Eigenspectrum. *EOF* pairs 1, 2 and 3, 4 as the basis of possible oscillatory components in the preprocessed wind velocity series.

**Fig 6:**
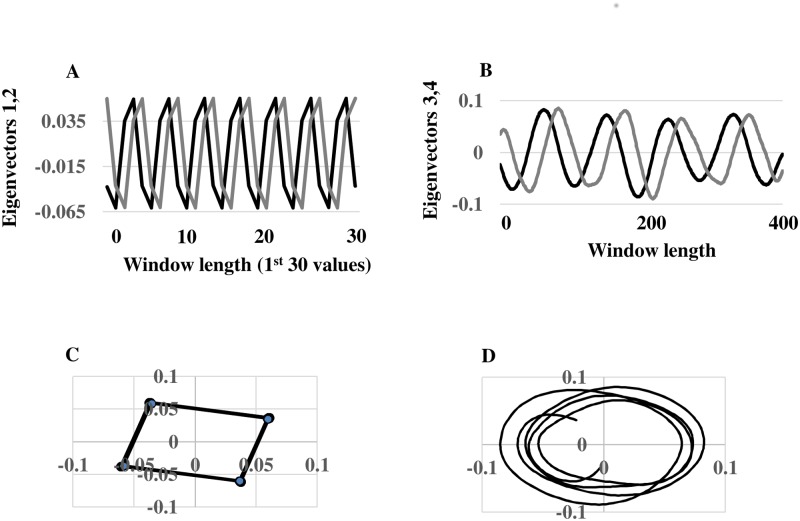
2^nd^ Stage Oscillations. (A) The graphs of the eigenvectors associated with *EOFs* 1 and 2 oscillate with identical frequency in phase quadrature. (B) The scatterplot of these eigenvectors results in a four-sided polygon reflecting a four-cycle of 6-hour blocks, i.e., a diurnal oscillation. (C) The graphs of the eigenvectors associated with *EOFs* 3 and 4 oscillate with identical frequency in phase quadrature. (D) The scatterplot of these eigenvectors gives a circular polygon reflecting a relatively low-frequency cycle of 100 six-hour blocks (25 days).

**Fig 7:**
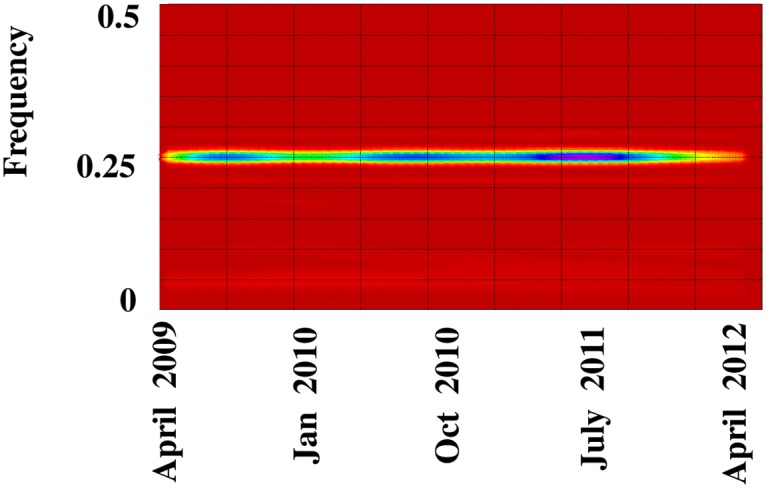
Signal Detection. The continuous wavelet spectrum of first-stage SSA residuals verifies stationary power at the dominant frequency of the diurnal oscillation.

**Fig 8:**
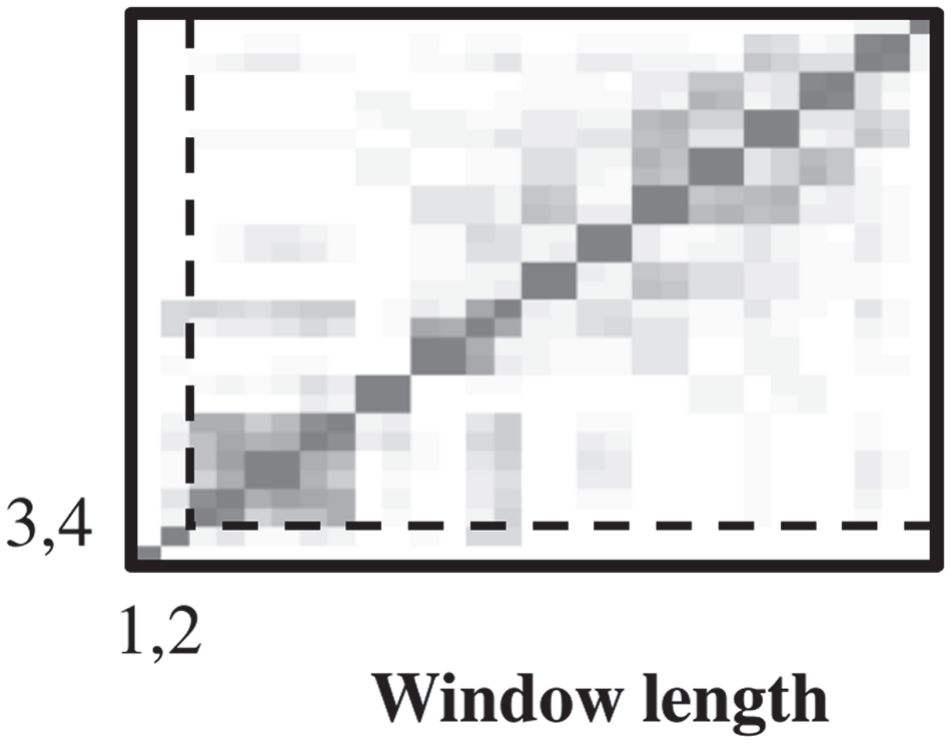
*w-Correlations*. The *w-correlation* matrix shows required statistical independence of *EOF* pairs 1, 2 and 3, 4. Lighter shaded boxes indicate low *w-correlations*.

A second application of SSA to 1^st^ stage residuals (i.e., *v*_*H*_ less the reconstructed trend) extracted diurnal and low-frequency 600-hour (= 25-day) oscillations. The eigenspectrum indicates potential harmonic oscillations associated with *EOF* pairs 1, 2 and 3, 4 (Fig. 5). The corresponding paired eigenvectors oscillate with diurnal and 100 six-hour (= 25 day) periods in phase quadrature, respectively (Figs. 6A, B). In addition, pairwise scatterplots of the paired eigenvectors are visually similar to scatterplots of sine and cosine functions oscillating at the same frequencies (Figs. 6C, D). In particular, the square appearance of the pairwise scatterplot associated with *EOFs* 1,2 displays a diurnal oscillation of four six-hour blocks (Fig. 6C). The circular-polygon appearance of the pairwise scatterplot associated with *EOFs* 3, 4 reflects the low-frequency 25 day oscillation (Fig. 6D). The continuous wavelet spectrum of 1^st^ stage residuals verifies stationary power at the frequency of the diurnal oscillation (0.25 Hz) (Fig. 7). The absence of time-localized power variations is consistent with spectral stationarity required by nonlinear dynamic analysis [19]. The *w-correlations* calculated between the time series’ reconstructed from *EOF* pairs 1, 2 and 3, 4 and those reconstructed from remaining *EOFs* are low (as demonstrated by lightly-shaded boxes below the dashed lines in Fig. 8), indicating required separability among groupings.

**Fig 9:**
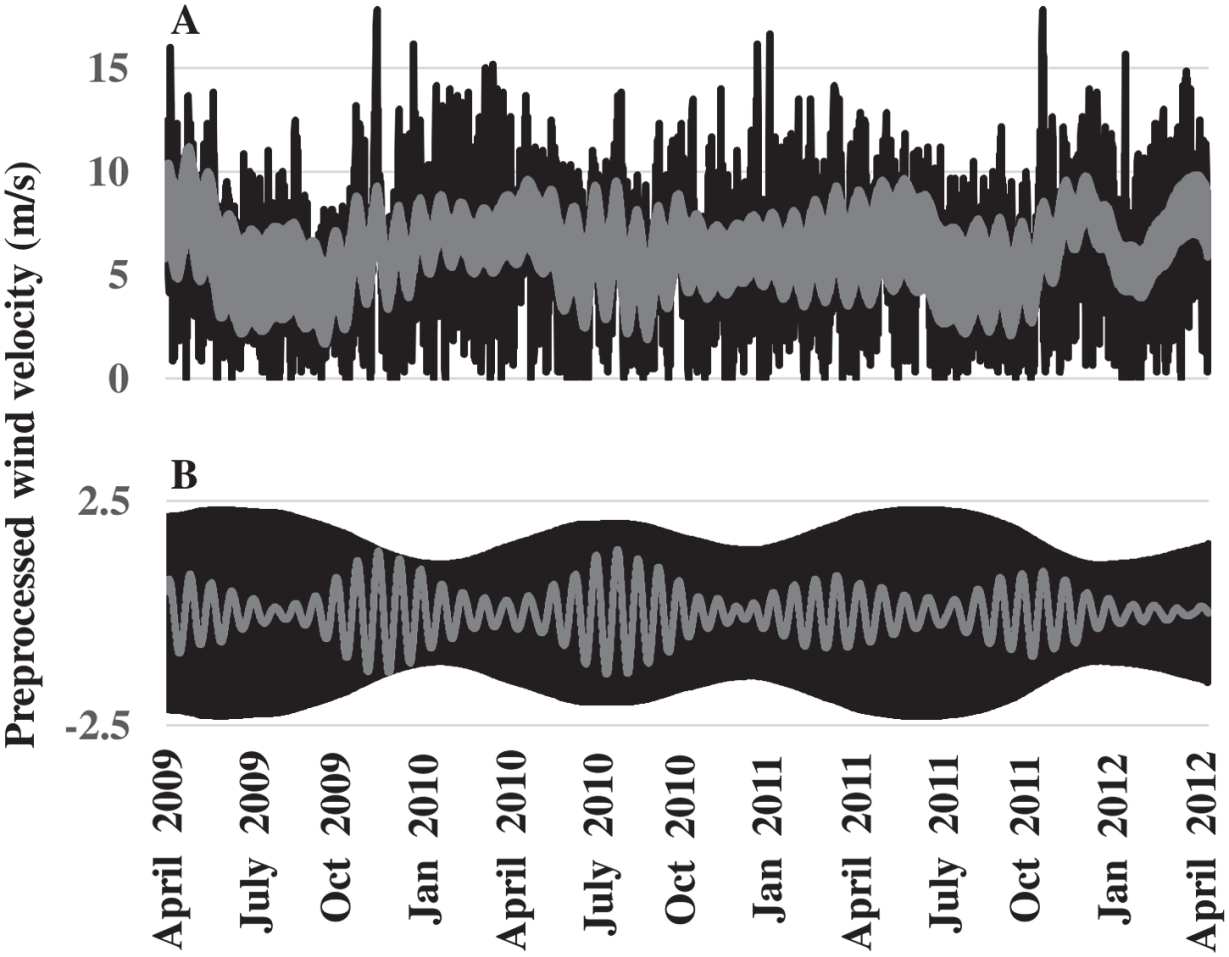
2^nd^ Stage SSA. (A) Plots of preprocessed wind-speed series (black line) and denoised series reconstruction (grey line). (B) Plots of extracted diurnal oscillation (black line) and 25-day oscillation (grey line).

The sum of reconstructed trend and oscillatory time series’ (without the unstructured-residual component) is plotted against the preprocessed wind speed record (*v*_*H*_) in Fig. 9A, and extracted diurnal (black line) and 25-day (grey line) oscillations are plotted in Fig. 9B. The denoised SSA reconstruction faithfully reproduces lower and higher frequency oscillatory behavior of *v*_*H*_, but not the full magnitudes of observed peaks/troughs. However, the objective of SSA is not to replicate time series observations exactly (this is possible by reconstructing a time series using all *EOFs*), but to reconstruct the time series as the composite of identified temporal patterns useful for prediction and explanation of persistent dynamic behavior. Compelling evidence for the quality of the SSA reconstruction is that the trend and two oscillatory components together account for about 85% of total variance in the preprocessed wind speed record, *v*_*H*._

### Phase Space Reconstruction

The phase space depicting wind speed dynamics for the project site was reconstructed from the SSA-reconstructed wind speed record with the trend and unstructured-residual components removed. (*v*_*SSA*_) Following the ‘time-delay embedding method’ [14,30–32], the multidimensionality of the real-world dynamic system was obtained by segmenting *v*_*SSA*_(*t*) into a sequence of *m* delay coordinate vectors, *v*_*SSA*_ *(t)*, *v*_*SSA*_(*t—d*), *v*_*SSA*_(*t—2d*), …, *v*_*SSA*_(*t—(m—1)d*) with delay *d*. These vectors are columns in a (*N-m+1*)×*m* ‘embedded data matrix’, where *N* is the length of the time series. The consecutive rows of this matrix are points along a trajectory in reconstructed phase space. The trajectory is a sampling portraying a ‘skeleton’ of the real-world attractor [13,19].*m ≥ 2n + 1* If, the reconstructed attractor shares key topological properties with a reconstruction in any coordinate system, where *n* is the (unobserved) dimension of the real-world attractor [19]. These properties include ‘correlation dimension’ and ‘Lyapunov exponent’. The correlation dimension measures the geometric dimension of the attractor, and also gives the minimum number of variables required to construct phase space [33]. The Lyapunov exponent measures the average rate at which initially close points on an exponentially diverge or converge; and consequently, indicates the extent to which the attractor exhibits sensitivity to initial conditions [30]. Strange attractors are characterized by low-dimensional fractal correlation dimensions and positive Lyapunov exponents [31].

**Fig 10:**
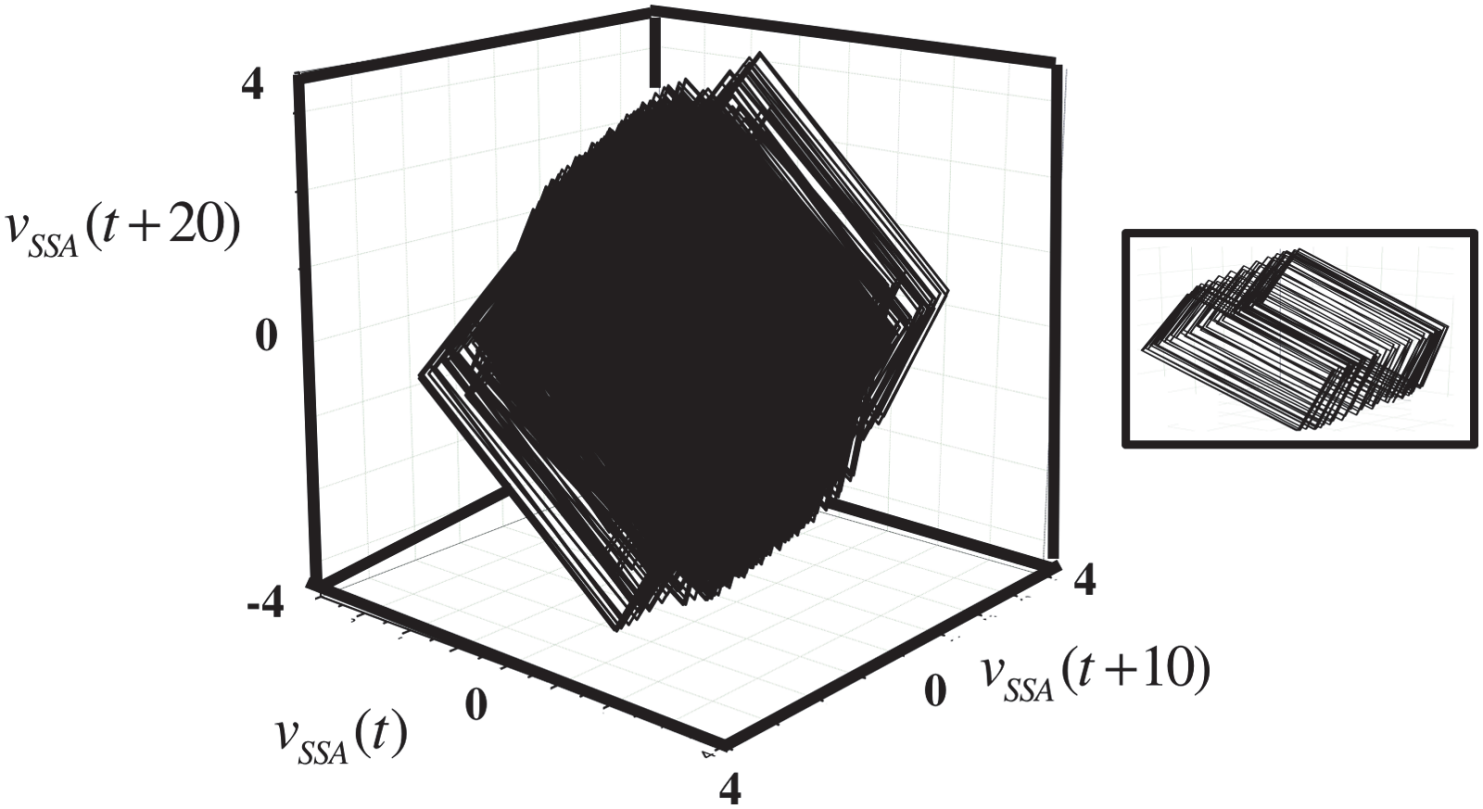
Phase Space Reconstruction. Wind speed dynamics evolve along a nonlinear low-dimension attractor reconstructed from lagged copies of the denoised and detrended SSA-reconstructed series. The attractor exhibits the dominant four-cycle of six-hour blocks giving the diurnal oscillation. The inset highlights the internal structure of the attractor by plotting only the first 200 points.

The attractor reconstructed from *v*_*SSA*_ displays the square shape characteristic of irregular diurnal oscillations each composed of four six-hour blocks (Fig. 10). The embedding dimension, *m* = 3, was selected using the conventional ‘false nearest neighbors’ method, and the delay, *d* = 10 hours, as the first minimum of the mutual information function [14]. The estimated correlation dimension is 2.35, giving evidence that the wind-speed attractor is low-dimensional. We did not calculate the Lyapunov exponent because of difficulties in computing reliable estimates from finite noisy records [20,31].

### Surrogate Data Testing

We applied surrogate data methods to test the null hypothesis that deterministic structure apparently displayed by the reconstructed wind-speed attractor is more likely the figment of a mimicking stochastic process [20,21,34]. Surrogate data vectors destroy patterns in observed data while preserving various statistical properties. Phase space is reconstructed from each surrogate vector, and selected topological properties are calculated and collected in frequency distributions. For each topological property, the mean (*μ*_*M*_) and standard deviation (*σ*_*M*_) of the surrogate distribution are used to compute a one-sample t-statistic (*t*_*tp*_) testing for statistical difference between attractors reconstructed from surrogates and that reconstructed from observed data:

$$t_{tp}\;=\;\frac{\mu_{M}-M}{SE_{M}}$$ (2)

where *M* is the property measured for the observed attractor, $SE_{M}\;=\;\sigma_{M}/\sqrt{N}$ is the standard error, and *N* is the length of the observed data. Statistically insignificant *t*-statistics indicate acceptance of the null hypothesis that detected structure in the ‘observed’ attractor is better attributed to stochastic behavior.

We generated one hundred surrogate data vectors for each of two conventionally-tested stochastic processes: (1) *aaft* (amplitude-adjusted Fourier transform) surrogates calculated as static monotonic nonlinear transformations of linearly filtered noise that preserve both the signal’s probability distribution and power spectrum [20,21,34]; and (2) *PPS* surrogates testing for the presence of a noisy limit cycle by preserving periodic trends in the signal while destroying chaotic structures [34].

We selected correlation dimension and predictive skill—a hallmark of deterministic structure—as discriminating properties [20,21,34,35]. Following state-space forecasting methods [35,36], points on the reconstructed and surrogate attractors were split into forecasting and validation bases. Initially, the nearest *m*+1 neighboring points to the final point in the forecast base, *D(T)*, were computed, advanced one time period, and averaged to forecast the first point in the validation base, *D(T+1)*, where *m* is the embedding dimension. We used a weighted-averaging simplex method [32]. At each step, the forecasting base was augmented by a point in the validation base until all points in the validation base (excepting the final point) had been predicted. The first coordinate of the predicted point *P(t)* in the time-delay state space is predicted wind speed, and the second and third coordinates are lagged predicted wind speeds. We compared the predictive skill of attractors constructed from surrogates against that of the observed attractor with the mean squared error (*mse*) [35]:

$$mse\;=\;\frac{1}{k-1}{\sum_{i=1}^{k-1}\left\|D_{T+i}-P_{T+i}\right\|}^{2}$$ (3)

which is squared Euclidean distance between points on the attractor and their predicted values in the validation base averaged over the number of rows in the validation base (*k*) less one.

We specified a two-tailed hypothesis test for correlation dimension to reject the null hypothesis for mean surrogate values significantly above or below the correlation dimension for the observed attractor. The null hypothesis is rejected for the set of critical significance levels *α_c_* satisfying:

$$\alpha_{c}\;\geq \;2\left(1-\Phi \left|t\right|\right)$$ (4)

where the right-hand side of the inequality is the *p*-value for a two-tailed test, Φ|*t*| is the CDF for the *t*-statistic with *N-1* degrees of freedom, and || is absolute value. We specified an upper-tailed hypothesis test for predictive skill to reject the null hypothesis only if the observed attractor predicts with higher skill (lower *mse*) than the battery of surrogate attractors on average. The null hypothesis is rejected for:

$$\alpha_{c}\;\geq \;1-\Phi (t)$$ (5)

where the right-hand side is the *p*-value for an upper-tailed test.

Surrogate data results reject the null hypotheses that the observed attractor’s deterministic structure is generated by the tested stochastic processes (Table 1). The *p*-values are zero to at least two decimal places for both discriminating measures.

**Table 1:** Surrogate Data Analysis.

Stochastic Process	Signal	Surrogates: Mean	Surrogates: St Dev	p-value
AAFT surrogates				
Correlation dimension	2.35	1.37	0.12	0.00
Mean prediction error	0.57	1.80	0.14	0.00
PPS surrogates				
Correlation dimension	2.35	0.71	0.07	0.00
Mean prediction error	0.57	2.55	0.70	0.00

### Wind Power Supply along Wind-Speed Attractor

**Fig 11:**
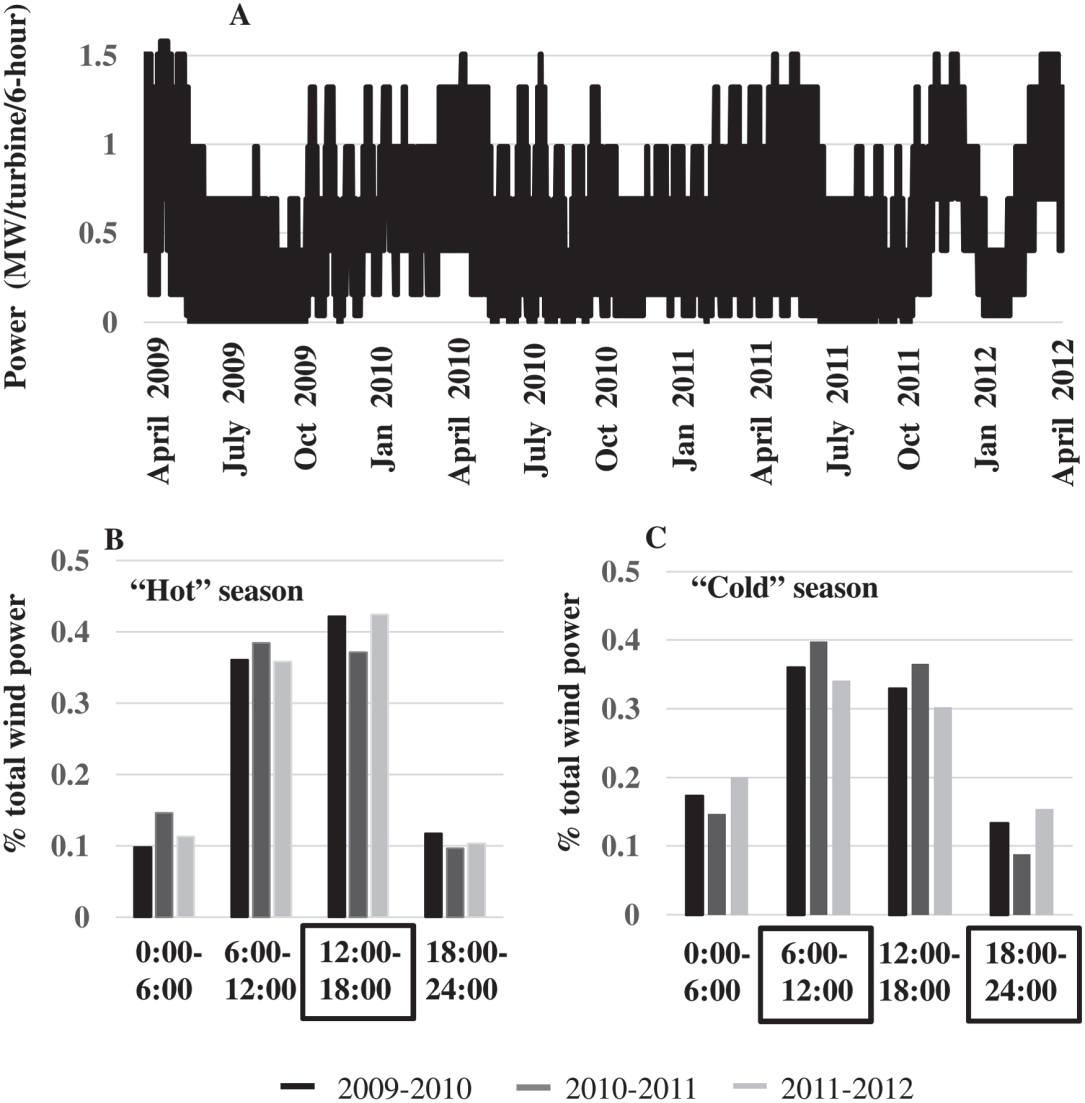
Wind power supply. (A) Wind power (*MW*) generated on the reconstructed attractor per turbine per 6-hour interval from April 2009 through March 2012. (B) The percentage of total wind power generated during “hot” season months (April through October). The largest percentage of wind power is generated during the peak afternoon demand period (12:00–18:00). (C) The percentage of total wind power generated during “cold” season months (November through March). The largest percentage of power is generated during the peak morning demand period (6:00–12:00), the lowest percentage is generated during the peak evening demand period (18:00–24:00).

Tabular values for the GE 1.6–100 turbine power curve [10] were used to compute electricity (*MW*) generated by the systematic wind-speed patterns defining the wind-speed attractor (Fig. 11A). The wind speeds used to calculate electricity in 6-hour blocks were the sum of the values taken from the first coordinate axis of the attractor, *v*_*SSA*_(*t*), and the reconstructed trend component. Wind power along the attractor is truncated at zero for wind speeds less than the cut-in speed of 3.5 *m/s* and greater than the cut-out speed of 26 *m/s*, and at the rated-power level of 1.6 *MW* for wind speeds between the rated-power speed of 12 *m/s* and the cut-out speed (Fig. 2B).

We first considered the extent to which natural diurnal wind speed patterns would allocate power to 6-hour blocks of peak daily demand (Fig. 2A) over the study period 2009–2012. During the “hot” season (April through October), the non-peak morning (6:00–12:00) and peak afternoon (12:00–18:00) time blocks would receive from 35–42% of wind power in the three years (Fig. 11B). During the “cold” season (November through March), the peak morning (6:00–12:00) and the non-peak afternoon (12:00–18:00) time blocks would receive from 30–40% of wind power in the three years, and the peak evening time block (18:00–24:00) would receive from 9–15% (Fig. 11C). We conclude that diurnal wind speed patterns would perform best in matching wind power to peak daily demand in the hot season, and to peak morning demand in the cold season. However, increased wind power penetration in the peak cold season evening interval would require greater reliance on costly grid-scale electricity storage.

**Fig 12:**
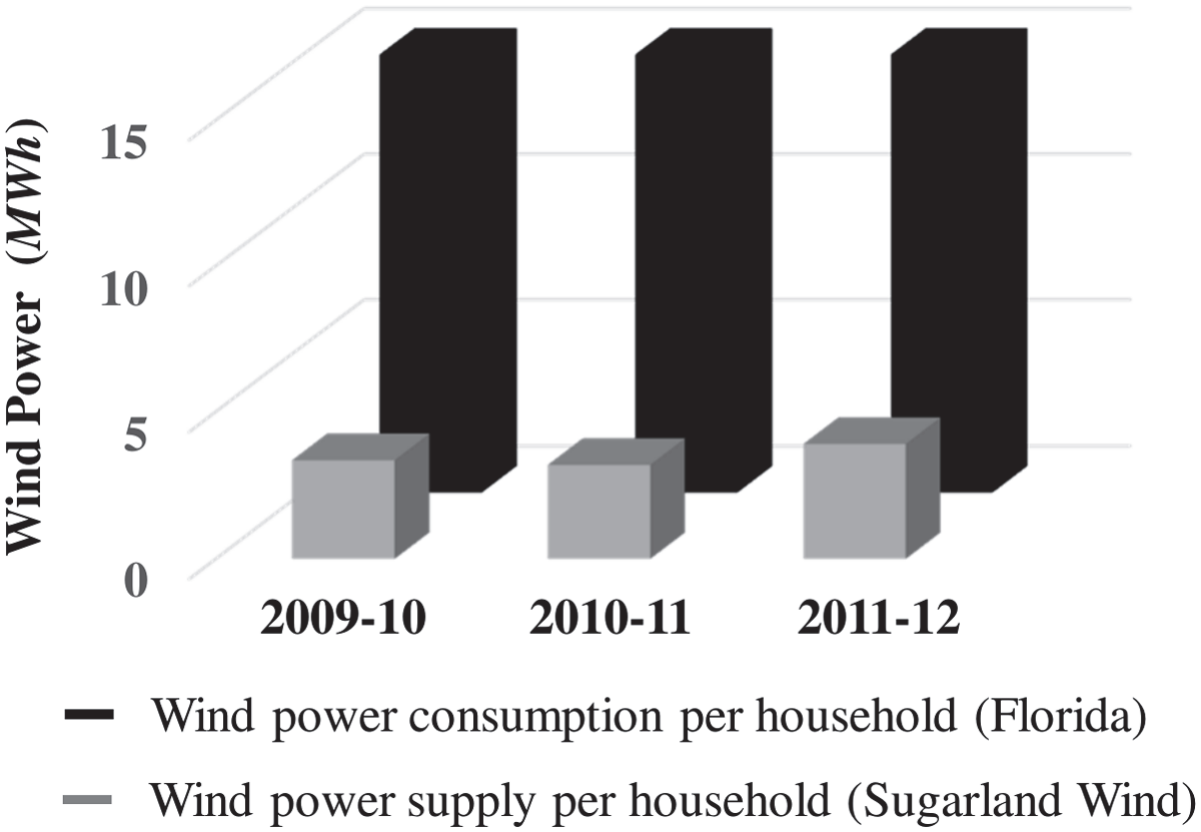
Sugarland Wind’s average annual supply rates per household. Wind power (*MWh*) generated along the reconstructed attractor for 114 turbines and 60,000 households planned for Sugarland Wind (grey boxes) supplies 22%–26% of average annual electricity consumption per household in Florida (black boxes) for 2009–2010, 2010–11, and 2011–12.

We next considered the extent to which wind power generated along the wind-speed attractor would satisfy average electricity consumption per household during the three years in our study 2009–2012. The total wind power (*MW hours*, *MWh*) generated for the 114 turbines planned for Sugarland Wind was calculated to be 201,470 (2009–10), 192,238 (2010–11), and 234,393 (2011–12). A resolution supporting Sugarland Wind predicted electricity for 60,000 Florida households [22]. This yields average supply rates per household for the project area of 3.36 (2009–10), 3.20 (2010–11), and 3.91 (2011–12) assuming that wind power generated off-peak were sold when generated or stored for reallocation to peak demand periods. Fig. 12 compares these figures to average consumption rates per household for Florida as a whole: 15.52 (2009–10), 16.12 (2010–11), and 15.13 (2011–12). Electricity sales per year in Florida are provided by the U.S. Energy Information Administration (2014) [37], and the number of Florida households 1990–2012 is obtained from Bureau of Economic and Business Research (2014) [38]. Wind power generated along the wind-speed attractor for the 60,000 households served by Sugarland Wind would supply about 22% (2009–10), 20% (2010–11), and 26% (2011–12) of their average annual consumption.

### Out-of-Sample Forecasting

**Fig 13:**
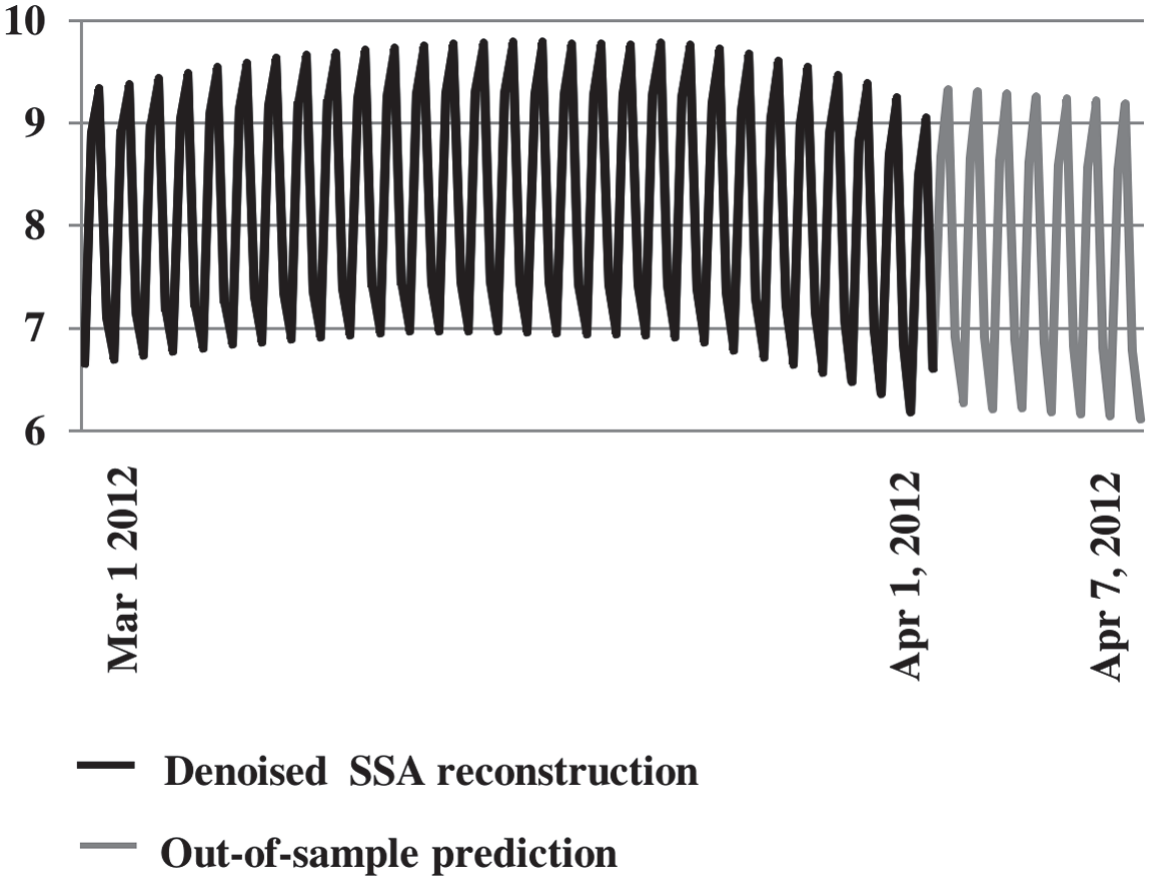
Out-of-Sample Forecasting (1 week). Out-of-sample forecasts successfully project reconstructed diurnal and lower-frequency oscillations.

State space forecasting methods described above were used to forecast wind power out-of-sample. The forecasting base included all points on the attractor. At each step, the predicted point was added to the attractor to predict the next point out-of-sample [35]. The week-long out-of-sample prediction of SSA-reconstructed wind speeds (*v*_*SSA*_ with reconstructed trend added) successfully projected diurnal and lower-frequency oscillations (Fig. 13).

## Discussion and Conclusions

The demand for electricity follows regular daily and seasonal patterns. The literature underscores that wind speeds also follow strong diurnal patterns, and that wind power penetration would increase if natural supply patterns coincided with demand patterns. We formulated a procedure to detect and characterize wind-power supply patterns in project evaluation. The procedure functioned well in the Sugarland Wind case study. Nonlinear dynamic methods succeeded in reconstructing a low-dimensional and nonlinear attractor from behavioral patterns in the historic wind-speed record. The reconstructed attractor is characterized by an expected strong diurnal oscillation and a fainter 25-day oscillation. Wind power generated along the reconstructed attractor generally matched well with peak daily demand in the hot season, and with peak morning demand in the cold season. It did not coincide well with peak evening demand in the cold season, indicating potential need for increased energy storage. Nonlinear forecasting with the reconstructed attractor succeeded in reproducing these oscillations outside of the sample.

A limitation of a nonlinear dynamics approach is that attractor reconstruction in wind-project evaluation is not a given, and can fail for a couple of major reasons [39]. For example, wind-speed dynamics in a project area may not be governed by a low-dimensional attractor, noisy or limited data may prevent an existing low-dimensional attractor from being detected, or observed data may not rest on the attractor. When nonlinear dynamic techniques fail to detect natural wind-power patterns, conventional stochastic approaches remain a viable alternative. However, we propose that project evaluators initially test for natural wind-power patterns in the data before presuming stochastic structures potentially falling short of real-world complexity.
